# Supplementary material for: Distances Between Extension Spaces of Phylogenetic Trees
Source: IEEE Trans Comput Biol Bioinform. Author manuscript; Available in PMC 2025 Aug 15. (PMC12356048; doi:10.1109/TCBBIO.2025.3526422)
Supplement: supp1-3526422 [file NIHMS2072649-supplement-supp1-3526422.pdf]

Supplementary Materials: Distances between Extension Spaces of Phylogenetic Trees

I. Algorithm to find BHV distances between orthant-specific extension spaces.

See Algorithm 1 for the proposed reduced gradient method to find distances between orthant-specific extension spaces.

```
Set initial values: ẋ_j^0 = Σ_i 1_{Ṁ[i,j]>0} × v̇_i / #{j : Ṁ[i,j] = 1}.
Define initial index sets: For each i, add the index j of the first column such that Ṁ[i,j] = 1 to D. Add all j' > j such that
Ṁ[i,j'] = 1 to F. Set N= ∅.
Initialize c_conj = 1. Set tolerance thresholds Tol1 and Tol2.
while global minimum not reached do
  Compute gradient ∇φ(ẋ_F^t) = ∇_F δ(ẋ^t) − Ṁ_F^⊤ Ṁ_D^{−⊤} ∇_D δ(ẋ^t)
  if ||∇φ(ẋ_F^t)||_∞ < Tol1 then
    Compute ḡ_N = ∇_N δ(ẋ^t) − Ṁ_N^⊤ Ṁ_D^{−⊤} ∇_D δ(ẋ^t).
    if ḡ_N ≥ 0 then
      stop while: global minimum has been reached.
    else
      Define N_p = {j ∈ N | ḡ_N[j] < 0}
      Update F = F ∪ N_p and N = N \ N_p
    end if
  end if
  Compute d_F^t = −∇φ(ẋ_F^t) + 1_{c_conj=1} ⟨∇φ(ẋ_F^t), ∇φ(ẋ_F^t) − ∇φ(ẋ_F^{t−1})⟩ / ||∇φ(ẋ_F^{t−1})||^2 d_F^{t−1}
  Set d_D^t = −Ṁ_D^{−1} Ṁ_F d_F^t and d_N^t = 0. Increase c_conj ← c_conj + 1
  Find τ_max = max{τ ≥ 0 | ẋ^t + τ d^t ≥ 0} and h(τ_max) = ⟨∇δ(ẋ^t + τ_max d^t), d^t⟩
  if h(τ_max) ≤ 0 then
    Set τ_0 = τ_max
  else
    Set τ_left = 0, τ_right = τ_max and τ_0 = (τ_left + τ_right)/2
    while |h(τ_0) = ⟨∇δ(ẋ^t + τ_0 d^t), d^t⟩| > Tol2 do
      if h(τ*) > 0 then set τ_right = τ* and τ* = (τ_left + τ_right)/2
      else set τ_left = τ* and τ* = (τ_left + τ_right)/2.
    end while
  end if
  if ẋ_j^t + τ_0 d_j^t = 0 for some j ∈ D ∪ F then
    Select j ∈ D ∪ F such that ẋ_j^t + τ_0 d_j^t = 0
    if j ∈ F then
      Set F = F \ {j} and N = N ∪ {j}
    else
      Select j' ∈ F such that Ṁ[i,j] = Ṁ[i,j'] = 1 for some index i
      Set D = D \ ({j} ∪ {j'}), F = F \ {j'} and N = N ∪ {j}
    end if
  end if
  Update ẋ^{t+1} = ẋ^t + τ* d_t
  if c_conj + 1 > 15 then c_conj = 1 else c_conj ← c_conj + 1
end while
return (T_1'(ẋ^t), T_2'(ẋ^t)) and sqrt(δ(T_1'(ẋ^t), T_2'(ẋ^t)))
```

Algorithm 1: A reduced gradient method to find BHV distances between orthant-specific extension spaces.

II. Proof of Lemma 4.2

Lemma 4.2. Given two trees T_1 and T_2 with leaf sets $\mathcal{L}_1, \mathcal{L}_2 \subseteq \mathcal{N}$ and an orthant pair $(O_1, O_2) \in C_{T_1}^{\mathcal{N}} \times C_{T_2}^{\mathcal{N}}$, the distance between the orthant-specific extension spaces equals the distance between their orthant-specific mutually restricted extension space, that is, $\inf\left\{d(T_1', T_2') \,\middle|\, (T_1', T_2') \in E_{T_1}^{O_1} \times E_{T_2}^{O_2}\right\} = \inf\left\{d(T_1', T_2') \,\middle|\, (T_1', T_2') \in \lceil E_{T_1}^{O_1} \times E_{T_2}^{O_2} \rceil\right\}$.

Proof. Since $\lceil E_{T_1}^{O_1} \times E_{T_2}^{O_2} \rceil \subseteq E_{T_1}^{O_1} \times E_{T_2}^{O_2}$, we need only show that for any pair $(T_1', T_2') \in E_{T_1}^{O_1} \times E_{T_2}^{O_2}$ there is a pair $(T_1^*, T_2^*) \in \lceil E_{T_1}^{O_1} \times E_{T_2}^{O_2} \rceil$ such that $d(T_1^*, T_2^*) \leq d(T_1', T_2')$. We will construct such a pair by first setting all inconsequential common edges to a proper length and all uncommon inconsequential edges to length zero.

We begin by defining (T_1^0, T_2^0) such that $\mathcal{O}(T_1^0) \subseteq O_1$ and $\mathcal{O}(T_2^0) \subseteq O_2$, $|s|_{T_1^0} = |s|_{T_1'}$ for all $s \in \mathcal{P}(O_1) \setminus \mathcal{P}(O_2)$, $|s|_{T_2^0} = |s|_{T_2'}$ for all $s \in \mathcal{P}(O_2) \setminus \mathcal{P}(O_1)$, and the conditions for mutual restriction hold for common edges $p \in \mathcal{P}(O_1) \cap \mathcal{P}(O_2)$:

$$
\begin{aligned}
|p|_{T_1^0} = |p|_{T_1'}, |p|_{T_2^0} = |p|_{T_2'} &\text{ when } \Psi_{\mathcal{L}_1}(p) \neq \emptyset \text{ and } \Psi_{\mathcal{L}_2}(p) \neq \emptyset \\
|p|_{T_1^0} = |p|_{T_2^0} = |p|_{T_2'} &\text{ when } \Psi_{\mathcal{L}_1}(p) = \emptyset \text{ and } \Psi_{\mathcal{L}_2}(p) \neq \emptyset \\
|p|_{T_2^0} = |p|_{T_1^0} = |p|_{T_1'} &\text{ when } \Psi_{\mathcal{L}_1}(p) \neq \emptyset \text{ and } \Psi_{\mathcal{L}_2}(p) = \emptyset \\
|p|_{T_1^0} = |p|_{T_2^0} = 0 &\text{ when } \Psi_{\mathcal{L}_1}(p) = \emptyset \text{ and } \Psi_{\mathcal{L}_2}(p) = \emptyset
\end{aligned}
$$

Since the lengths of the uncommon internal splits are unchanged from (T_1', T_2') to (T_1^0, T_2^0), the support of the path space will be the same for both pairs (common edges lengths do not influence the support for the geodesic [1, Section 4]). For those edges that are inconsequential in one or both trees, the difference in length drops to zero; it remains the same for edges that are consequential for both (for such an edge p, $|p|_{T_1'} - |p|_{T_2'} = |p|_{T_1^0} - |p|_{T_2^0}$). We denote by K^0 the common edges (including external edges) that are consequential for both extension spaces (i.e. $s \in K^0 \subseteq K = C \cup H$ when $\Psi_{\mathcal{L}_1}(s) \neq \emptyset$ and $\Psi_{\mathcal{L}_2}(s) \neq \emptyset$)

$$
\begin{aligned}
d(T_1^0, T_2^0) &= \left|\left|\left(||A_1||_{T_1^0} + ||B_1||_{T_2^0}, \ldots, ||A_k||_{T_1^0} + ||B_k||_{T_2^0}, \left(|s|_{T_1^0} - |s|_{T_2^0}\right)_{s \in K}\right)\right|\right| \\
&= \left|\left|\left(||A_1||_{T_1^0} + ||B_1||_{T_2^0}, \ldots, ||A_k||_{T_1^0} + ||B_k||_{T_2^0}, \left(|s|_{T_1^0} - |s|_{T_2^0}\right)_{s \in K^0}, 0\right)\right|\right| \\
&= \left|\left|\left(||A_1||_{T_1'} + ||B_1||_{T_2'}, \ldots, ||A_k||_{T_1'} + ||B_k||_{T_2'}, \left(|s|_{T_1'} - |s|_{T_2'}\right)_{s \in K^0}, 0\right)\right|\right| \\
&\leq d(T_1', T_2').
\end{aligned}
$$

Although all common edges in the tree pair (T_1^0, T_2^0) hold the conditions for mutual restrictions, some of the uncommon edges between both trees may not. Define the inconsequential edges in T_1^0 that are not common with T_2^0 by $(p_1^1, \ldots, p_{r_1}^1)$, and the inconsequential edges in T_2^0 not common with T_1^0 by $(p_1^2, \ldots, p_{r_2}^2)$. For each $i = 1, 2$, define $T_i^j = T_i^{(j-1) \perp p_j^i}$ the projection of T_i^{j-1} towards the face of O_i defined by the length of p_j^i being equal to zero. By repeatedly applying Lemma 4.1, we have

$$
d(T_1^0, T_2^0) \geq d(T_1^1, T_2^0) \geq \ldots \geq d(T_1^{r_1}, T_2^0) \geq d(T_1^{r_1}, T_2^1) \geq \ldots \geq d(T_1^{r_1}, T_2^{r_2}).
$$

Thus, $d(T_1^{r_1}, T_2^{r_2}) \leq d(T_1', T_2')$, and by construction, $(T_1^{r_1}, T_2^{r_2}) \in \lceil E_{T_1}^{O_1} \times E_{T_2}^{O_2} \rceil$. □

III. Algorithm 1: additional details

A. Reduced gradient directions

The direction of change will be based on the gradient of δ at the current point $\dot{\mathbf{x}}^t$ when all partial derivatives in (11) are well-defined, and a subgradient otherwise. To compute this (sub)gradient, we first find the support for the geodesic from $T_1'(\dot{\mathbf{x}}^t)$ to $T_2'(\dot{\mathbf{x}}^t)$, and use its support to determine the entries of the (sub)gradient. We then focus on the reduced gradient within the current facet, which is $\nabla\varphi(\mathbf{x_F}) = \nabla_{\mathbf{F}} f(\mathbf{x}) - A_{\mathbf{F}}^\top \left[A_{\mathbf{D}}^{-1}\right]^\top \nabla_{\mathbf{D}} f(\mathbf{x})$ for the general case. Due to the structure of $\dot{\mathbf{M}}$, the partial derivative corresponding to a free variable $\dot{\mathbf{x}}_j^t$, $j \in \mathbf{F}$, is $\frac{\partial\varphi(\dot{\mathbf{x}}_{\mathbf{F}})}{\varphi \dot{\mathbf{x}}_j^t} = \nabla_j \delta(\dot{\mathbf{x}}) - \nabla_{j'} \delta(\dot{\mathbf{x}})$, where $j' \in \mathbf{D}$ is the only index in the dependent variable set such that $\dot{\mathbf{M}}[i, j] = \dot{\mathbf{M}}[i, j'] = 1$.

There are multiple ways to determine a good direction of change for the free variables at $\dot{\mathbf{x}}_{\mathbf{F}}^t$. We employed the conjugate gradient method [2, Section 5.5] because of its simplicity and the potential gain in efficiency. In this method, the main driver for the direction of change is the (sub)gradient at the current point, but after the first iteration a correction is added to increase efficiency. The correction loses its advantages and new directions become inefficient after many iterations [2, Section 5.5.2], thus we re-initialize the correction regularly. The direction of change $\mathbf{d}_{\mathbf{F}}^t$ for the free variables is computed by

$$
\mathbf{d}_{\mathbf{F}}^t = \begin{cases} -\nabla\varphi(\dot{\mathbf{x}}_{\mathbf{F}}^t) & \text{if } c_{\text{conj}} = 1 \\ -\nabla\varphi(\dot{\mathbf{x}}_{\mathbf{F}}^t) + \frac{\langle \varphi(\dot{\mathbf{x}}_{\mathbf{F}}^t), \varphi(\dot{\mathbf{x}}_{\mathbf{F}}^t) - \varphi(\dot{\mathbf{x}}_{\mathbf{F}}^{t-1}) \rangle}{\left|\left|\varphi(\dot{\mathbf{x}}_{\mathbf{F}}^{t-1})\right|\right|^2} \mathbf{d}_{\mathbf{F}}^{t-1} & \text{if } c_{\text{conj}} > 1, \end{cases} \tag{1}
$$

for a counter c_{conj}. In our method, c_{conj} is re-initialized (reset to 1) every time a new facet is reached (i.e. $\mathbf{F}$ is re-defined and $\mathbf{d}_{\mathbf{F}}^{t-1}$ is no longer of the same dimension) or when $c_{\text{conj}} > 15$. The threshold of 15 was recommended by [2, Page 248], and we found it to work well in our setting in practice. Given the direction of change for the free variables $\mathbf{d}_{\mathbf{F}}^t$, we can find the direction of change for the dependent variables as $\mathbf{d}_{\mathbf{D}}^t = -\dot{\mathbf{M}}_{\mathbf{D}}^{-1} \dot{\mathbf{M}}_{\mathbf{F}} \mathbf{d}_{\mathbf{F}}^t$ and for the null variables as $\mathbf{d}_{\mathbf{N}}^t = 0$.

TABLE I
THE COMPLETE LEAF SET $\mathcal{N}$ FOR THE *ftsA* AND *dinB* GENE TREES.

Species	Domain	*ftsA* tree	*dinB* tree
Actinomyces odontolyticus	Bacteria	No	Yes
Fusobacterium nucleatum	Bacteria	Yes	Yes
Pseudomonas pelagia	Bacteria	Yes	Yes
Bacteroides fragilis	Bacteria	Yes	Yes
Candidatus Saccharibacteria TM7x	Bacteria	Yes	No
Sphingomonas hengshuiensis	Bacteria	Yes	Yes
Parcubacteria SG8-24	Bacteria	Yes	No
Vibrio scophthalmi	Bacteria	Yes	Yes
Candidatus Lokiarchaeota CR4	Archea	No	Yes
Candidatus Odinarchaeota LCB4	Archea	No	Yes

B. Selecting step sizes

Given a non-zero direction of change $\mathbf{d}^t$, we now discuss finding the best next point on the line segment $\dot{\mathbf{x}}^t + \tau\mathbf{d}^t$ such that both $\tau \geq 0$ and $\dot{\mathbf{x}}^t + \tau\mathbf{d}^t \geq 0$. Let τ_{max} be the maximum value of τ such that $\dot{\mathbf{x}}^t + \tau\mathbf{d}^t \geq 0$, which is finite because $\dot{\mathbf{M}}(\dot{\mathbf{x}}^t + \tau\mathbf{d}^t) = \dot{\mathbf{v}}$ implies $\dot{\mathbf{M}}\mathbf{d}^t = 0$, and thus some entry of $\mathbf{d}^t$ is negative. Thus, we are looking for $\tau \in [0, \tau_{\text{max}}]$ that minimizes $\delta(\dot{\mathbf{x}}^t + \tau\mathbf{d}^t)$.

Using the chain-rule, the derivative of $\delta(\dot{\mathbf{x}}^t + \tau\mathbf{d}^t)$ with respect to τ is

$$h(\tau) = \frac{\partial\delta(\dot{\mathbf{x}}^t + \tau\mathbf{d}^t)}{\partial\tau} = \langle\nabla\delta(\dot{\mathbf{x}}^t + \tau\mathbf{d}^t), \mathbf{d}^t\rangle. \quad (2)$$

The function $\tau \mapsto \delta(\dot{\mathbf{x}}^t + \tau\mathbf{d}^t)$ is convex and by construction of $\mathbf{d}^t$, $h(0) < 0$. From this, we employ a derivative-based bisection method to reach the minimum of this function. We start by checking the value of $h(\tau_{\text{max}})$. If $h(\tau_{\text{max}}) \leq 0$, then we have reached the minimum at $\tau_0 = \tau_{\text{max}}$. Otherwise, we search for the value $\tau_0 \in [0, \tau_{\text{max}}]$ such that $h(\tau_0) = 0$ as follows

1) Initialize $\tau_{\text{left}} = 0$ and $\tau_{\text{right}} = \tau_{\text{max}}$.
2) Take $\tau^* = \frac{\tau_{\text{left}} + \tau_{\text{max}}}{2}$.
3) Evaluate $h(\tau^*)$:
   - If $h(\tau^*) = 0$, return τ^*.
   - If $h(\tau^*) < 0$, update $\tau_{\text{left}} = \tau^*$ and return to step 2.
   - If $h(\tau^*) > 0$, update $\tau_{\text{right}} = \tau^*$ and return to step 2.

In practice, we do not require $h(\tau^*) = 0$ exactly; instead, we require $|h(\tau^*)|$ to be below a threshold. We find that $|h(\tau^*)| < 10^{-16}$ works well in practice. This threshold can be altered in our software via the flag `-Tol2`. After finding τ_0, we select the next point as $\dot{\mathbf{x}}^{t+1} = \dot{\mathbf{x}}^t + \tau_0\mathbf{d}^t$.

C. Thresholds for convergence

Our iterative algorithm is guaranteed to converge to stationary points with zero (sub)gradients. However, in practice, we employ a tolerance threshold to find a solution that is sufficiently close to a stationary point in a finite number of iterations. Specifically, before computing a new direction of change, we test if we have reached the global minimum by checking if every entry in $\nabla\varphi(\dot{\mathbf{x}}_{\mathbf{F}})$ is less than a given threshold `Tol1`. In practice, we find that choosing this threshold to be 10^{-8} produces good performance. The user can alter this value using the flag `-Tol1`.

REFERENCES

[1] M. Owen and J. S. Provan, "A fast algorithm for computing geodesic distances in tree space," *IEEE/ACM Transactions on Computational Biology and Bioinformatics*, vol. 8, no. 1, pp. 2–13, 2011.
[2] A. P. Ruszczyński, *Nonlinear optimization*. Princeton, N.J: Princeton University Press, 2006.

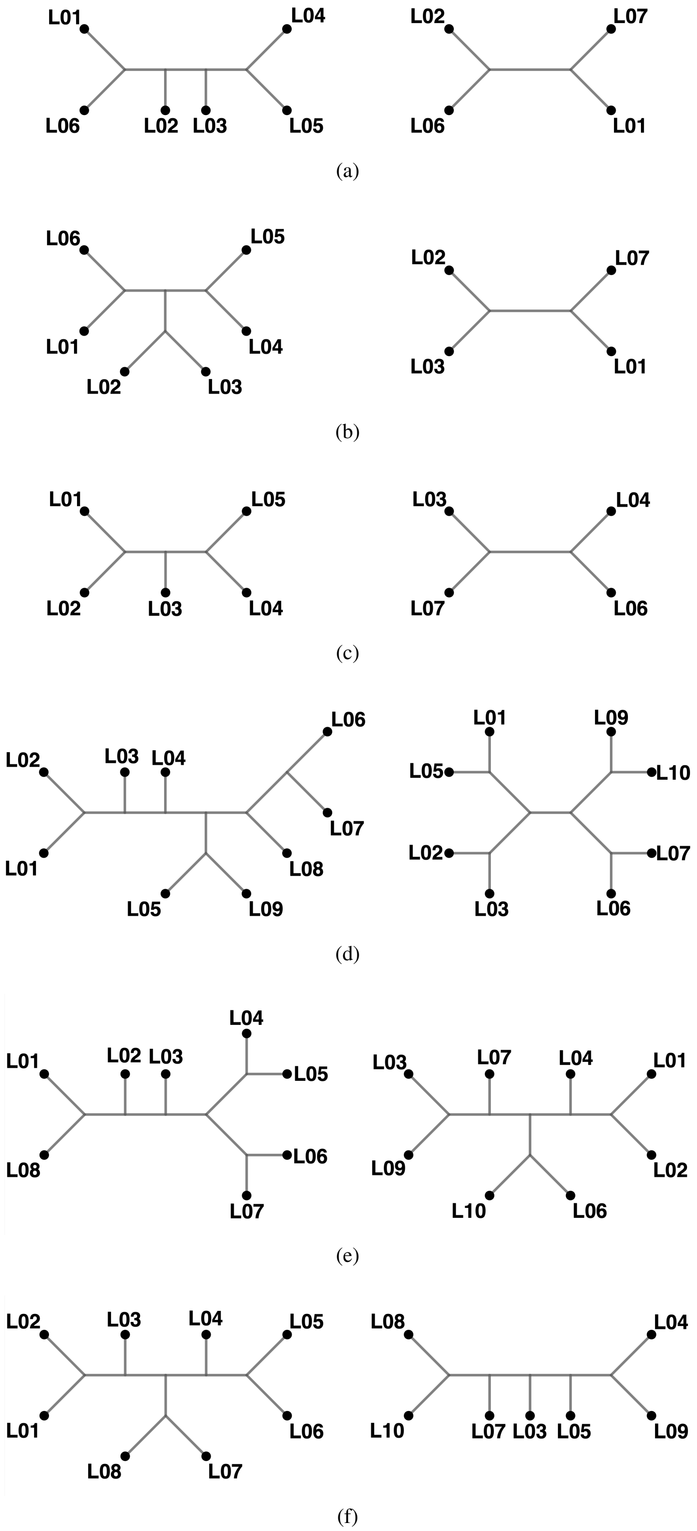

Fig. 1. The six pairs of topologies used in our runtime analysis (Section 5). The edge lengths for these trees were randomly generated, and can be found at github.com/statdivlab/ExtnSpaces_supplementary.git.

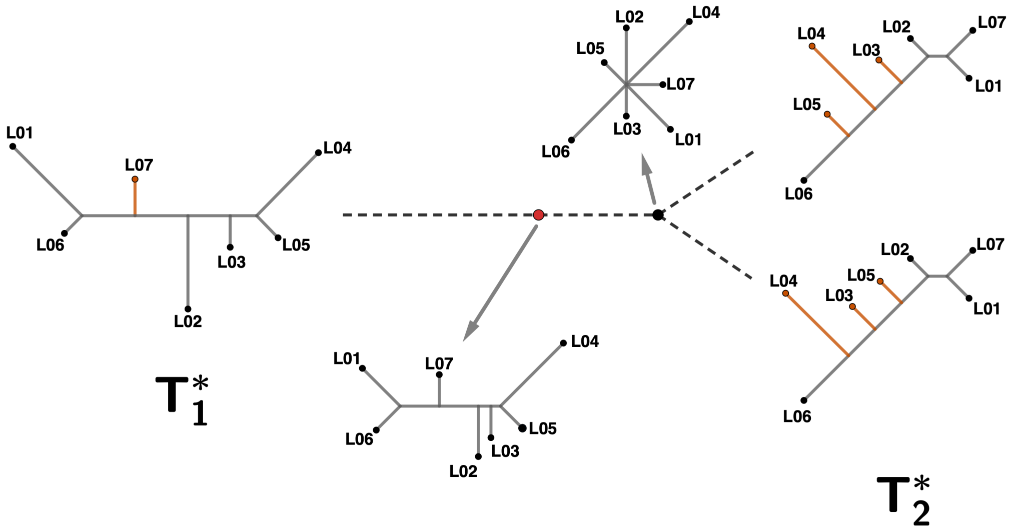

Fig. 2. An example demonstrating how non-unique optimal pairs can yield unique midpoints (simulation study, setting (a) with a bimodal distribution of edge lengths). Inconsequential edges are shown in orange. The tree T_1^* (left) is identical for all optimal pairs. The two trees labeled T_2^* (right) represent two of optimal six possibilities in the extension space of T_2. The remaining pairs are derived by regrafting exterior edges to **L03**, **L04**, and **L05** in same positions with identical lengths, but in different orders. The dashed line shows the geodesic within an optimal pair. Note that these paths are identical from T_1^* until the tree where all internal edges reach zero length (black dot). The geodesic midpoint is marked in red.

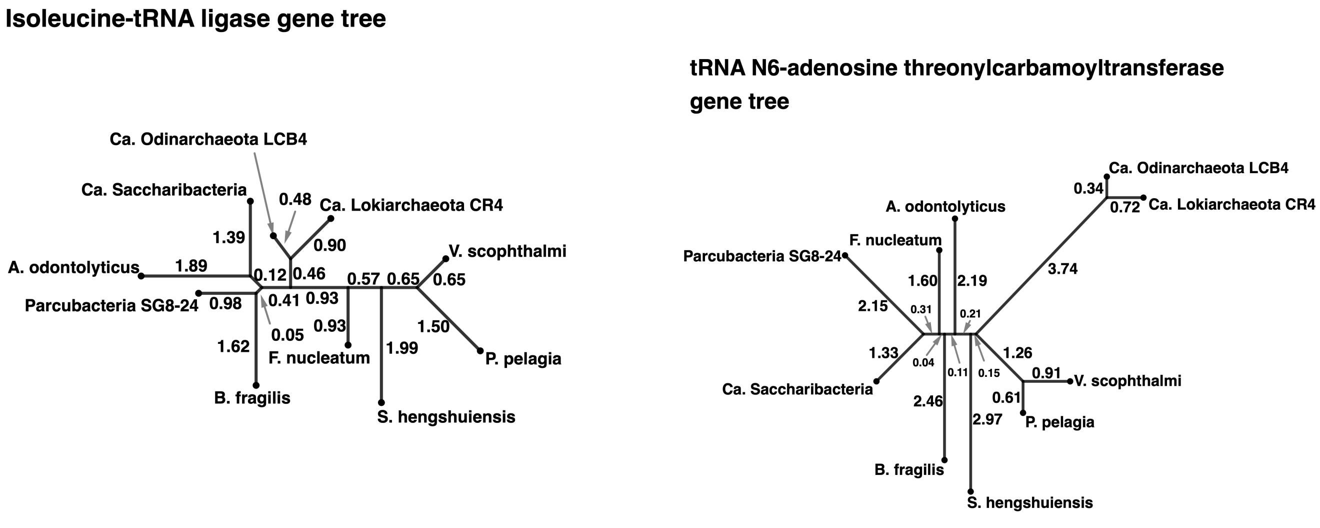

(a) (b)

Fig. 3. The estimated evolutionary history of the *IleRS* (a) and *TsaD* (b) genes.
